# Post-operative re-bleeding in patients with hypertensive ICH is closely associated with the CT blend sign

**DOI:** 10.1186/s12883-017-0910-6

**Published:** 2017-07-06

**Authors:** Guofeng Wu, Zhengkui Shen, Likun Wang, Shujie Sun, Jinbiao Luo, Yuanhong Mao

**Affiliations:** 1 The Affiliated Hospital of Guizhou Medical University, No. 28, Guiyijie Road, Liuguangmen, Guiyang City, 550004 Guizhou Province People’s Republic of China; 2 Shanghai Clinical Research Centre of Chinese Academy of Sciences, No.966, Huaihai Middle Road, Shanghai City, 200233 People’s Republic of China; 3 Guangzhou First People’s Hospital, No.1, Panfu Road, Guangzhou City, 510000 People’s Republic of China

**Keywords:** Intracerebral haemorrhage, Minimally invasive surgery, Blend sign, Computed tomography, Post-operative re-haemorrhage

## Abstract

**Backgrounds:**

Intracranial post-operative re-haemorrhage is an important complication in patients with hypertensive intracerebral haemorrhage (ICH). The purpose of the present study was to determine the value of the computed tomography (CT) blend sign in predicting post-operative re-haemorrhage in patients with ICH.

**Methods:**

A total of 126 patients with ICH were included in the present study. All the patients underwent standard stereotactic minimally invasive surgery(MIS) to remove the ICH within 24 h following admission. There were 41 patients with a blend sign on initial CT and 85 patients without a blend sign on the initial CT. Multivariable logistic regression analyses were performed to assess the relationship between the presence of the blend sign on the non-enhanced admission CT scan and post-operative re-haemorrhage.

**Results:**

Post-operative re-haemorrhage occurred in 24 of the 41 patients with the blend sign, and in 9 of the 85 patients without the blend sign. The incidence of re-haemorrhage was significantly different between the groups. The multivariate logistic regression analysis demonstrated that the initial Glasgow coma scale score (*p* = 0.002) and blend sign (*P* < 0.00) on the initial CT scan are independent predictors of post-operative re-haemorrhage. The sensitivity, specificity, and positive and negative predictive values of the blend sign for predicting post-operative re-haemorrhage were 72.7, 81.7, 58.5 and 89.4%, respectively.

**Conclusions:**

The presence of the blend sign on the initial CT scan is closely associated with post-operative re-haemorrhage in patients with ICH who undergo stereotactic MIS.

## Background

Hypertensive intracerebral haemorrhage (ICH) results in long-term disability or death in a large proportion of afflicted patients [1]. Despite the known pathophysiological benefits of haemostasis and clot removal, ICH lacks an effective medical or surgical treatment [2]. Minimally invasive surgery (MIS) for ICH evacuation has shown promising results in recent years [2–5]. However, early intracranial postoperative re-haemorrhage is a great challenge and devastating neurological complication. Stereotactic aspiration of ICH improves the general condition of the patients, promotes improvement of consciousness, and decreases the incidence of pneumonia, but it may induce re-haemorrhage [6]. Previous studies have reported that the re-haemorrhage occurred in 40% of patients treated within 4 h and 12% of patients treated within 12 h after the onset of ICH. Further, a relationship between the postoperative re-haemorrhage and mortality was apparent [7]. Postoperative re-haemorrhage in patients with hypertensive ICH after MIS is both a major clinical problem and a possible target for medical intervention. The incidence of re-haemorrhage was 10.0% in patients who underwent MIS and 15.4% in those who underwent conventional craniotomy. In any case, the ability to predict and observe postoperative re-haemorrhage is of great clinical importance. If imaging markers for predicting the postoperative re-bleeding could be identified and easily used, it would help guide clinical practice. Haematoma growth after acute hypertensive ICH is a detrimental event that is associated with progressive neurological deterioration and poor outcomes [8]. Investigators have reported several imaging markers for predicting haematoma growth or enlargement in patients with hypertensive ICH. The hypodensity in the haematoma, blend sign, and irregularity of the haematoma were found to be closely related to early haematoma growth or enlargement [9, 10]. However, there was no evidence of preoperative haematoma growth, which would represent an increased postoperative re-bleeding risk for the minimally invasive aspiration of spontaneous intracerebral haemorrhage [11]. Previous studies have demonstrated that the computed tomography angiography(CTA)spot sign is associated with more intraoperative bleeding, more postoperative re-haemorrhage, and larger residual ICH volumes in patients undergoing haematoma evacuation for spontaneous ICH [12]. Because patients with re-haemorrhage tend to have liver dysfunction and haemorrhagic tendency, patients with liver dysfunction and haemorrhagic tendencies should be identified as being at risk of post-operative re-haemorrahage [6].

The CT blend sign can be easily identified on regular non-enhanced CT and is highly specific for predicting haematoma growth [9, 10]. Little is known about the relationship between the blend sign on the initial CT scan and postoperative re-haemorrhage after MIS. We speculate that they are associated with postoperative re-haemorrhage in patients with hypertensive ICH who underwent MIS. The objective of the present study was to determine the clinical value of the CT blend sign in predicting postoperative re-haemorrhage in patients with hypertensive ICH who underwent MIS.

## Methods

### Patients

The present study was approved by the ethics committee of the Affiliated Hospital of Guizhou Medical University and performed in compliance with the WMA Declaration of Helsinki - Ethical Principles for Medical Research Involving Human Subjects. Informed consent was obtained from the authorized representative of each patient. For patients who had the ability to give the informed consent, informed consent was obtained from both the patient and the authorized family member.

Patients with hypertensive ICH who were admitted to The Affiliated Hospital of Guizhou Medical University between September 2015 and October 2016 were included in our study. Hypertensive ICH was confirmed on a non-enhanced CT scan showing parenchymal haemorrhage.

The inclusion criteria were as follows: patients with hypertensive ICH in the basal ganglia, cerebellum, thalamus or cerebral lobe. The patients were candidates for stereotactic MIS and over 18 years of age.

Patients with ICH due to head trauma, brain tumour, aneurysm, or malformation, those receiving anticoagulant therapy and/or antiplatelet drugs, or those that had secondary ICH from haemorrhagic transformation of a brain infarction, were excluded from the study. Patients with ICH located in the brainstem and those who were not suitable for surgery were also excluded from our study.

The patients were diagnosed by baseline CT scan within 12 h after the onset of symptoms. The MIS was performed at 24 h after admission. The follow-up CT scan was performed at least twice (two-three times) in the following 3 days after surgery. Demographic information and time to baseline and follow-up CT scans were recorded for each patient.

Based on the inclusion criteria, a total of 126 patients with hypertensive ICH were included in the present study. The mean age of the patients was 58.83 ± 13.05 years (age range, 31–87 years). The average baseline ICH volume was 33.35 ± 22.68 ml. The average GCS was 10.73 ± 3.03. There were 41 patients with the blend sign on the initial CT scan and 85 patients without the blend sign. The baseline clinical characteristics of patients with and without the blend sign on CT are listed in Table 1. No statistically significant differences in terms of age, sex, hypertension, diabetes mellitus, smoking, and alcohol drinking were observed between the groups.

**Table 1:** Comparison of basic data between patients with and without the blend sign on baseline CT

Characteristics	Blend sign positive (*n* = 41)	Blend sign negative (*n* = 85)	*P* value
Mean age, yr. ($\overline{\mathrm{x}}$ ±s)	56.44 ± 14.85	59.98 ± 12.01	0.155
Sex, male (%)	28(68.3)	64(75.3)	0.052
Smoking (%)	19(46.3)	41(48.2)	0.266
Hypertension (%)	27(65.9)	47(55.3)	0.506
Diabetes mellitus (%)	2(4.9)	1(1.2)	0.313
Alcohol consumption (%)	20(48.8)	39(45.9)	0.578
Systolic pressure ($\overline{\mathrm{x}}$±s)	177.59 ± 26.62	171.78 ± 28.65	0.145
Diastolic pressure ($\overline{\mathrm{x}}$±s)	102.56 ± 16.71	97.92 ± 16.98	0.151
Time to baseline CT ($\overline{\mathrm{x}}$±s)	10.93 ± 2.84	11.74 ± 2.63	0.790
Baseline ICH volume ($\overline{\mathrm{x}}$±s)	33.68 ± 21.57	33.19 ± 22.32	0.190
Glasgow coma scale score ($\overline{\mathrm{x}}$±s)	9.83 ± 3.38	11.16 ± 2.77	0.020
Ruptured into the ventricle (%)	16(39.0)	47(55.3)	0.006
NIHSS on admission ($\overline{\mathrm{x}}$±s)	14.12 ± 4.62	13.38 ± 4.13	0.391

CT indicates computed tomography and ICH indicates intracerebral haemorrhage

### Imaging analysis

**Fig. 1:**
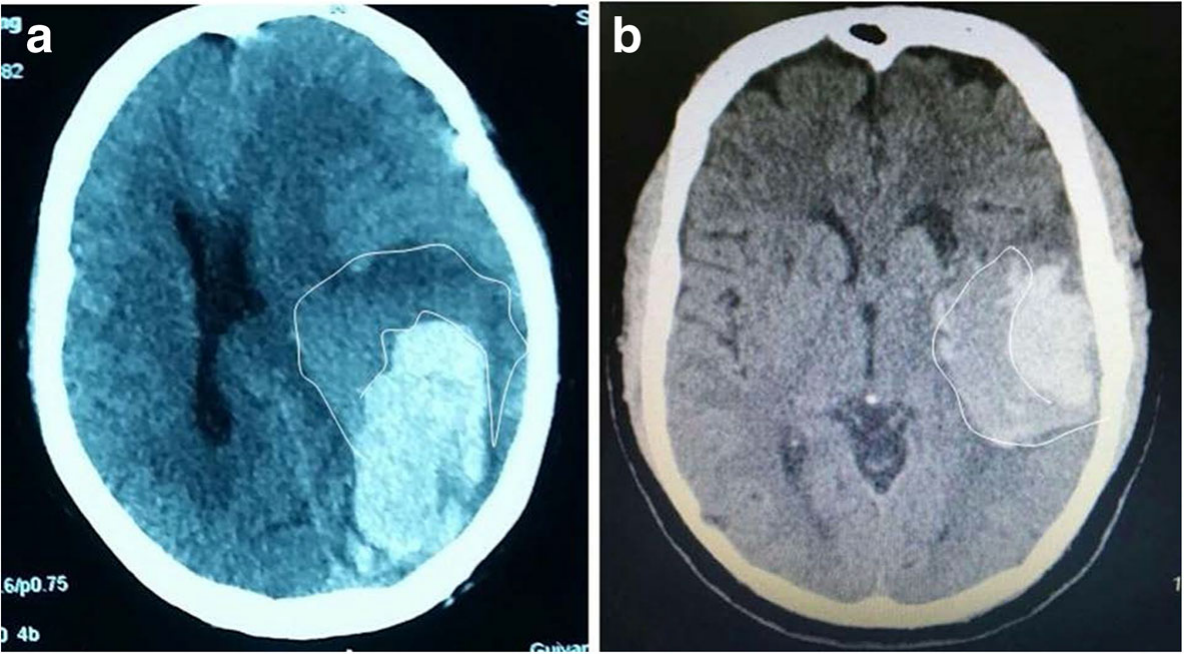
Blend sign on initial CT scan. The blend sign consists of two parts with apparently different CT attenuations. **a** The blend sign in patients with cerebral lobe haemorrhage. **b** The blend sign in patients with basal ganglion haemorrhage

The admission and follow-up CT scans were performed using standard clinical parameters with axial 3-mm-thick sections. The images were obtained and stored for further evaluation. The haemorrhage locations were classified as the basal ganglia, thalamus, lobe, brain stem, and cerebellum. Based on the criteria used in several large clinical studies on ICH, we defined intracranial postoperative re-haemorrhage as an increase in the haematoma volume of >33% compared to the previous postoperative CT scan (on which the ICH volume had decreased significantly after MIS), or reappearance of hyperdensity in the focal region at the follow-up CT scan after it was removed completely following surgery. Two experienced neuroimaging experts, who were blinded to the clinical information of the patients, acted as reviewers and independently evaluated the presence of the blend sign based on a recently proposed definition [9, 10]. Discrepancies about the presence of the blend sign were settled by joint discussion between the readers. The imaging features of the typical blend sign are illustrated in Fig. 1.

### Minimally invasive procedures for ICH evacuation

**Fig. 2:**
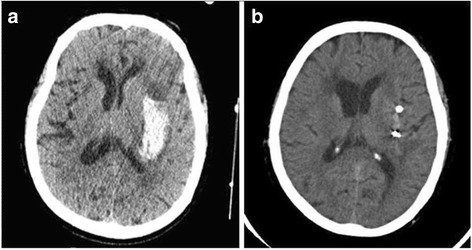
Hematoma changes after minimally invasive surgery. **a** The hyper-density on brain CT indicates pre-operative ICH in the left basal ganglion. **b** The ICH was removed by the minimally invasive surgery

The MIS for the ICH evacuation was the same procedure that was used in our previously published studies [13, 14]. Briefly, each patient underwent a repeat CT scan and was then transferred to the operating suite. The stereotactic instrument was fixed on the patient’s skull. Using the CT scan, the puncture position was selected, and the distance from the skin level to the desired location of the puncture needle tip was determined. Under the guidance of the stereotactic instrument, the skull was punctured by a 3-mm-diameter needle (type LY-1) under the driving force of an electrical drill. Then, the LY-1 type puncture needle was inserted slightly into the haematoma. Removing the needle core, the liquid part of the haematoma was aspirated with a 10 -ml syringe that was connected to the LY-1 type needle system. Subsequently, the haematoma cavity was flushed with sterile saline 2 to 3 times using a high-pressure jet-washing device. The patients were transferred to the intensive care unit after removing the location framework and stereotactic apparatus. The LY-1 type puncture needle system was removed after brain CT confirmed that the haematoma was either completely or nearly completely evacuated (Fig. 2). Generally, the postoperative follow-up CT scan was performed on the first day (the first postoperative CT) and the third day (the second postoperative CT) after surgery. Some patients needed a third or even a fourth postoperative follow-up CT scan.

### Statistical analysis

All statistical analyses were performed using a commercially available software package (SPSS, Version 22.0). Categorical data are presented as proportions and continuous variables are presented as $\overline{\mathrm{x}}$±s. The demographic, clinical, and radiological characteristics were compared between patients with and without intracranial postoperative re-haemorrhage using the Fisher exact test and Student’s *t* tests, as appropriate. *P* < 0.05 was considered statistically significant. The independent association between the CT blend sign and significant intracranial re-haemorrhage was evaluated using multivariable logistic regression. Receiver–operator analysis was performed to assess the value of the blend sign in predicting re-haemorrhage. The interobserver reliability of these signs was assessed by calculating the κ values. The κ values were categorized as follows: κ = 0.21 to 0.4, 0.41 to 0.6, 0.61 to 0.8, and 0.81 to 1 indicate low, moderate, substantial, and excellent agreement between observers, respectively. A κ = 1 indicates total agreement between the observers.

## Results

### Interobserver agreement of the blend sign

Forty-one patients out of the 126 were found to have the blend sign on the initial CT. Discrepancies were observed in only three patients between the two neuroimaging experts and were settled between them. Interobserver agreement for identifying the blend sign was excellent between the two readers.

## The haematoma growth prior to surgery

All the ICH patients received a repeat CT scan before surgery to calculate the target coordinates of the haematoma. Eight patients, including five patients with the blend sign and three patients without the blend sign, displayed haematoma growth according to the previously reported criteria.

### ICH evacuation and the postoperative CT follow-up

Using the CT scan from before surgery, we calculated a mean ICH volume of 33.68 ± 21.57(ml) in patients with the blend sign and 33.19 ± 22.32(ml) in patients without the blend sign. There was no significant difference in the ICH volume between the groups.

The postoperative follow-up CT scan was performed on the first day (the first postoperative CT) and the third day (the second postoperative CT) after surgery. Some patients needed a third or even a fourth postoperative follow-up CT. After a CT scan revealed that the ICH was mostly or completely evacuated the LY-1 type puncture needle system was removed. On the third day after the procedures, the average volume of the haematoma was 3.56 ± 1.50 ml (with a clearance rate of 93%) in patients with blend sign and 3.40 ± 1.65 ml (with a clearance rate of 92%) in the patients without the blend sign. No significant difference in the postoperative ICH volume was observed between the two groups. These results suggest that a minimally invasive technique can successfully evacuate the ICH.

A total of 33 patients with postoperative re-haemorrhage were found in the group of 126 patients who underwent MIS. One hundred and ten patients received postoperative follow-up CT twice. Eleven patients received follow-up CT three times. The other five patients received a fourth postoperative CT scan. The second postoperative follow-up CT found re-haemorrhage in 30 patients. In three patients re-haemorrhage was found by a third postoperative follow-up CT. Seventeen patients with large postoperative re-haemorrhage received a second surgery.

### Baseline characteristics of patients with postoperative re-haemorrhage

**Fig. 3:**
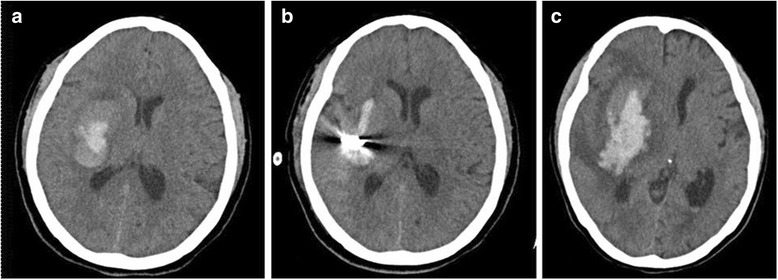
Post-operative re-haemorrhage in patients with blend sign. **a** The hyper-density on brain CT indicates pre-operative ICH in the right basal ganglion. The hyperdensity is composed of two parts with apparently different CT attenuations. **b** The ICH was removed with a minimally invasive procedure. **c** The hyper-density indicates postoperative re-haemorrhage in the same brain area as for the original hematoma

The average ICH volume after re-bleeding, as revealed by a repeat follow-up CT, was 26.30 ± 13.42 ml (from 8 ml to 60 ml) for the 33 patients with postoperative re-haemorrhage. The postoperative re-haemorrhage volume was significantly increased compared to that on the previous postoperative follow-up CT. The incidence of re-haemorrhage was 26.19% in the patients who underwent MIS. The baseline clinical characteristics of patients with and without the postoperative re-haemorrhage are listed in Table 2. There were significant differences in the baseline haematoma volume (*P* < 0.05), the Glasgow coma scale score (*P* < 0.05) and blend sign on initial CT (*P* < 0.05) between the patients with and without post-operative re-haemorrhage. These results suggest that the blend sign and disease condition were associated with post-operative re-haemorrhage. No statistically significant differences were observed between the groups in terms of age, sex, hypertension, diabetes mellitus, smoking, and alcohol drinking. The post-operative re-haemorrhage in patients with blend sign is illustrated in Fig. 3.

**Table 2:** Comparisons between the basic data between patients with and without re-haemorrhage

Characteristics	Re-haemorrhage group (*n* = 33)	Non-re-haemorrhage group (*n* = 93)	*P*
Mean age, yr. ($\overline{\mathrm{x}}$±s)	56.63 ± 15.20	59.77 ± 16.40	0.251
Sex, male (%)	23(69.70)	69(72.00)	0.534
Smoking (%)	18(54.50)	42(43.80)	0.281
Hypertension (%)	21(63.60)	53(55.20)	0.423
Diabetes mellitus (%)	1(3.00)	2(2.00)	0.774
Alcohol consumption (%)	17(51.50)	42(43.80)	0.468
Systolic pressure ($\overline{\mathrm{x}}$±s)	176.67 ± 27.30	174.39 ± 28.60	0.665
Diastolic pressure ($\overline{\mathrm{x}}$±s)	102.30 ± 17.60	98.82 ± 17.32	0.448
Time to baseline CT ($\overline{\mathrm{x}}$±s)	10.61 ± 3.19	11.78 ± 1.68	0.718
Baseline ICH volume ($\overline{\mathrm{x}}$±s)	25.15 ± 18.96	36.50 ± 23.84	0.045
Blend sign on baseline CT (%)	24(72.70)	17(17.70)	0.000
Ruptured into the ventricle (%)	13(39.40)	50(52.10)	0.318
Glasgow coma scale score ($\overline{\mathrm{x}}$±s)	11.64 ± 2.77	10.41 ± 3.07	0.045
Time to surgical procedures ($\overline{\mathrm{x}}$±s)	29.85 ± 28.54	20.84 ± 17.87	0.097
NIHSS on discharge ($\overline{\mathrm{x}}$±s)	17.06 ± 12.35	11.78 ± 9.18	0.026

CT indicates computed tomography and ICH indicates intracerebral haemorrhage

### The relationship between the CT blend sign and the post-operative re-haemorrhage

**Fig. 4:**
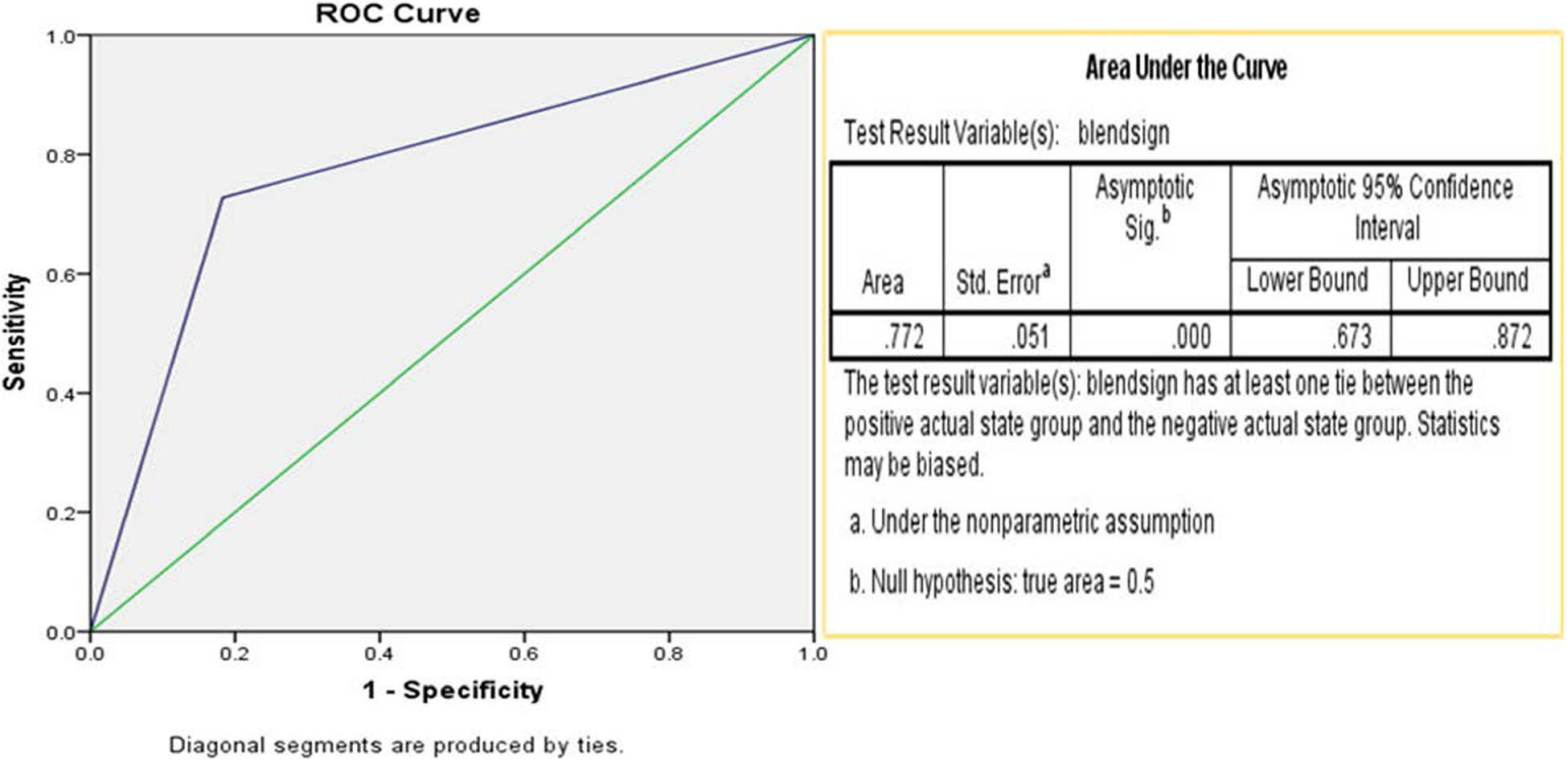
Receiver-operating characteristic (ROC) curve using a binary definition of postoperative re-haemorrhage showing an area under the curve = 0.772 and *P* < 0.05

Post-operative re-haemorrhage was found in 24 of the 41 patients with the blend sign (58.54%) and in 9 (10.59%) of the 85 patients without the blend sign on initial CT. A significant difference was observed in the incidence of re-haemorrhage between the groups. Of the 33 patients with post-operative re-haemorrhage, 24 patients (72.70%) had the blend sign on initial CT. However, of the 93 patients without re-haemorrhage, only 17 patients (17.70) had the blend sign on initial CT. The sensitivity, specificity, and positive and negative predictive values of the blend sign for predicting post-operative re-haemorrhage were 72.70, 81.70, 58.50, and 89.40%, respectively. The receiver–operator analysis was performed and confirmed the value of the blend sign in predicting postoperative re-haemorrhage (Fig. 4).

Univariate analysis of the predictors demonstrated that the Glasgow coma scale score (GCS), initial ICH volume and blend sign were associated with the incidence of post-operative re-haemorrhage. The GCS and blend sign, which were significant in the univariate logistic regression, were retained, but the baseline ICH volume was removed from the multivariate logistic model. The multivariate logistic regression analysis demonstrated that the GCS and presence of the blend sign on the baseline CT scan are independent predictors of post-operative re-haemorrhage (Table 3).

**Table 3:** Multivariate analysis of predictors for postoperative re-haemorrhage

Variables	Odds ratio	95% confidence Interval	*P* Value
Baseline ICH volume	1.022	0.994-1.050	0.132
Blend sign on baseline CT	42.365	10.487-171.348	0.000
Glasgow coma scale score	0.678	0.531-0.866	0.002

CT indicates computed tomography and ICH indicates intracerebral haemorrhage

## Discussions

Minimally invasive aspiration followed by thrombolysis has emerged as a promising strategy for modifying the neurological outcome of patients with ICH [3, 4, 11]. Post-operative re-haemorrhage remains a great clinical problem. Post-operative re-haemorrhage is associated with progressive neurologic deterioration and poor outcomes. The ability to predict and observe postoperative re-haemorrhage after MIS for ICH evacuation is of great clinical importance.

In the present study, we aimed to determine whether the initial CT blend sign is associated with post-operative re-haemorrhage in patients with ICH who underwent stereotactic MIS. Based on the initial CT scan, 41 patients with the blend sign on the initial CT and 85 patients without the blend sign on CT were included in our study. All patients underwent standard stereotactic minimally invasive aspiration followed by thrombolysis in the 24 h after the onset of symptoms. We analysed the difference in the re-haemorrhage incidence between the blend sign and non-blend sign groups to determine the association between the incidence of re-haemorrhage and presence of the imaging markers. The results showed that the incidence of re-haemorrhage was 58.54% (24/41) in patients with the blend sign and 10.59% (9/85) in patients without the blend sign on CT. A significant difference in the incidence of re-haemorrhage was observed between the blend sign and the non-blend sign group. Univariate analysis suggested that the blend sign, haematoma volume and GCS were associated with post-operative re-haemorrhage. Further multivariate analysis was performed to observe the value of these markers in predicting post-operative re-haemorrhage after the MIS. The results showed that the blend sign and GCS were independent predictors for post-operative re-haemorrhage in our small size sample study. However, the positive predicative value of the blend sign for the post-operative re-haemorrhage was low, although the sensitivity was significantly higher than that found by Li Qi [9, 10]. In Li′s study, the blend sign was used to predict natural haematoma growth, which was very different from the post-operative re-haemorrhage after the MIS. This might be the reason for the difference between the positive predictive value in our study and that reported in the literature [9, 10]. Additionally, sample size might be a reason for the difference in the positive predictive value between the two studies.

One of the most dangerous complications following minimally invasive stereotactic surgery for ICH evacuation is post-operative re-haemorrhage. The incidence of postoperative re-haemorrhage varies greatly in different studies [4, 5, 15, 16]. In our study, the incidence of postoperative re-haemorrhage in patients without the blend sign on initial CT was similar to that reported previously [15, 16]. However, the incidence of postoperative re-haemorrhage was significantly higher in patients with the blend sign on initial CT. The mechanism by which the blend sign induces postoperative re-bleeding is unclear.

Investigators have found that several imaging markers could predict haematoma growth [12, 17, 18]. The CT angiography spot sign indicates bleeding complications during and after surgery for ICH [19]. Patients with liver dysfunction and haemorrhagic tendencies easily develop postoperative re-bleeding [6]. However, few imaging markers are involved in predicting postoperative re-haemorrhage in patients with ICH who undergo MIS. Our study demonstrated that the blend sign could be an independent predictor for postoperative re-bleeding after MIS for ICH. Thus, patients with the blend sign on initial CT should be identified and measures for preventing postoperative re-bleeding should be enhanced in these patients. The blend sign is closely associated with early haematoma growth. Hematoma growth in patients with the blend sign on initial CT was 24/29 with a rate of approximately 71% [9]. In our study, the postoperative re-haemorrhage rate was 26.19% in total and 58.54% in 41 patients with the blend sign. Therefore, the blend sign may be useful for selecting patients for future surgical trials.

Postoperative re-haemorrhage after minimally invasive neurosurgery might be associated with many factors, such as surgical procedures, time from onset to surgery and abnormal coagulation mechanisms. The effect of the time interval on the postoperative re-bleeding was ruled out as the time to baseline CT was approximately 10 h and the time to the surgical procedures was about 24 h. Haematoma growth was most frequently seen in the first 6 h after onset. The influence of abnormal coagulation mechanisms on the postoperative re-bleeding was less likely as patients with anticoagulant therapy and antiplatelet therapy were excluded from the present study. The most probable factor leading to re-haemorrhage was blood vessel damage following the surgical procedures. However, this possibility has been excluded in the present study because the procedure was guided by a stereotactic apparatus and performed by the same skilled neurosurgeon. Post-operative re-haemorrhage occurred 1 or 2 days after the haematoma was nearly completely removed following the surgical procedures. If the re-haemorrhage were induced by the procedure itself, it would have occurred during the surgery. In addition, the incidence of re-bleeding in patients without the blend sign was similar to those reported previously. The time interval between the onset of symptoms and the initial brain CT scan is an important factor for predicting haematoma enlargement. However, it was less likely to influence our observation in the present study, as the baseline time intervals between the re-haemorrhage and non-re-haemorrhage groups were not different.

There are some limitations in our study. Because the sample size was small, we were unable to observe the relationship between postoperative re-bleeding and other imaging markers, such as the CTA spot sign. We were unable to rule out the possibility that postoperative re-bleeding was due to other factors. The ICH volume based on each postoperative repeated CT was not reported. Haematoma growth in the blend sign and non-blend sign groups was not analysed in detail prior to surgery. We did not analyse the influence of the pharmacotherapy on postoperative re-bleeding because all patients received the same pharmacotherapy, although surgery did differ. We did not compare the incidence of re-bleeding between the patients who received MIS versus conservative therapy. Further studies are required to elucidate the mechanism of the blend sign formation.

## Conclusion

In conclusion, our study suggests that the CT blend sign might be a predictor of postoperative re-bleeding in patients with ICH who underwent MIS. The CT blend sign might be useful for identifying patients with ICH who are likely to experience postoperative re-bleeding. Greater attention should be paid to procedures that prevent postoperative re-bleeding in patients with the blend sign on initial CT.

## Abbreviations

CT: Computed tomography
CTA: Computed tomography angiography
GCS: Glasgow coma scale
ICH: Intracerebral haemorrhage
MIS: Minimally invasive surgery
